# Supplementary material for: Human Embryonic Stem Cell-Derived Immature Midbrain Dopaminergic Neurons Transplanted in Parkinsonian Monkeys
Source: Cells. 2023 Nov 30;12(23):2738. doi: 10.3390/cells12232738 (PMC10706241; doi:10.3390/cells12232738)
Supplement: Supplementary file 1 [file cells-12-02738-s001.zip › cells-2702092-supplementary.pdf]

**Supplementary Figures**

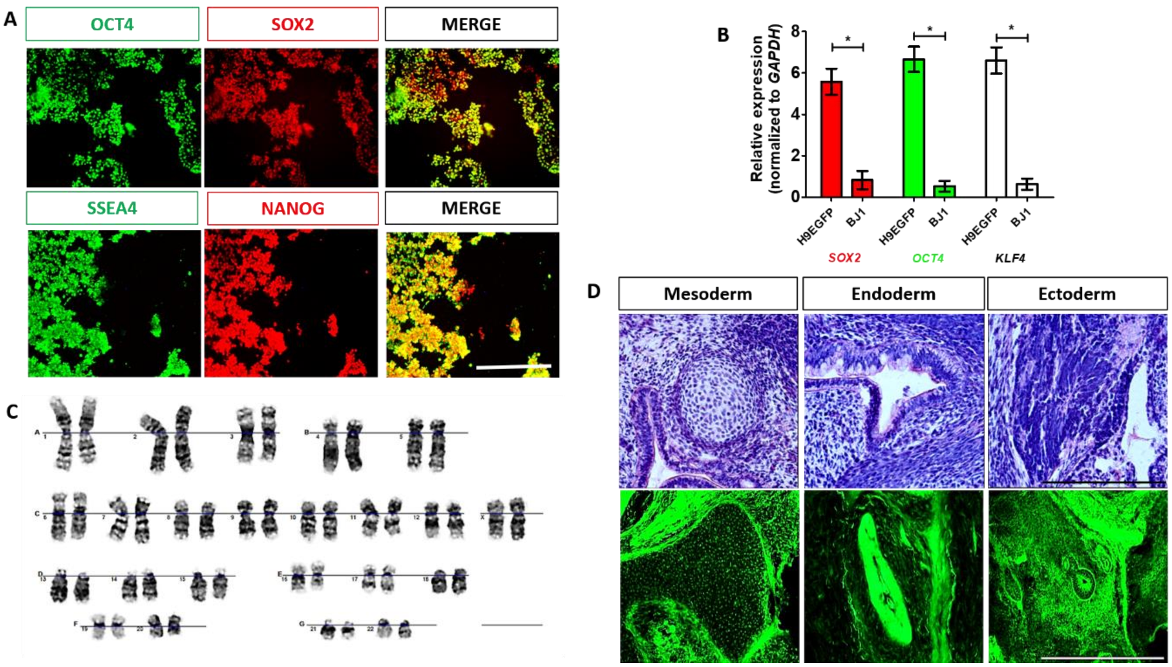


**Supplementary Figure 1.- Pluripotency of cell lines H9 and H9-EGFP. A)** Immunocytochemical analysis on H9 cell line. Scale bar, 200 µm. **B)** Gene expression, by RT-qPCR, for *SOX2*, *OCT4*, and *KLF4* on H9-EGFP cell line. Negative controls are BJ1 human fibroblasts. **C)** H9-EGFP cell line presents a normal female karyotype (46XX). **D)** Teratoma formation after H9-EGFP subcutaneous inoculation where cells representative of the three embryonic germ layers can be identified by hematoxylin and eosin staining (upper panel). The lower panel shows adjacent slices to observe EGFP. Mean ± SEM; *$P$ < 0.05, n=3 independent experiments. Scale bar, 300 µm.

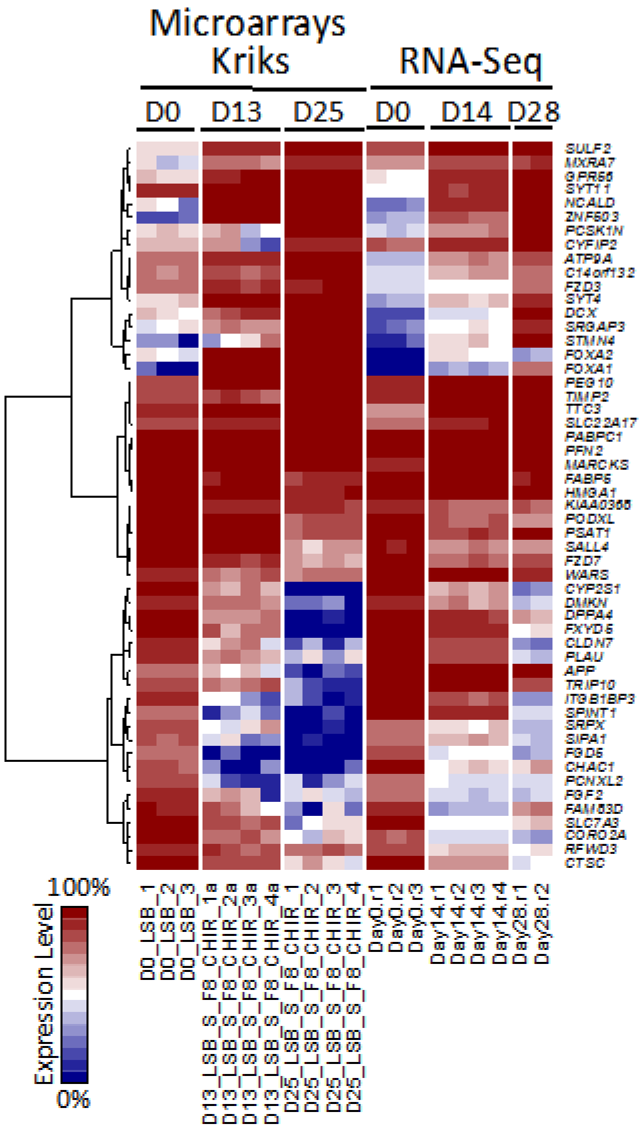


**Supplementary Figure 2.- Comparison of expression data at similar time points from previous work with our RNA-Seq results.** The microarray results from Kriks et al. [12] were aligned with the reads of the same genes found in our sequencing experiments [40]. Although the times are not the same (day 13 vs day 14 and day 25 vs day 28), the expression patterns are similar, indicating that our differentiation proceeded as reported for the floor-plate induction of dopamine neurons.

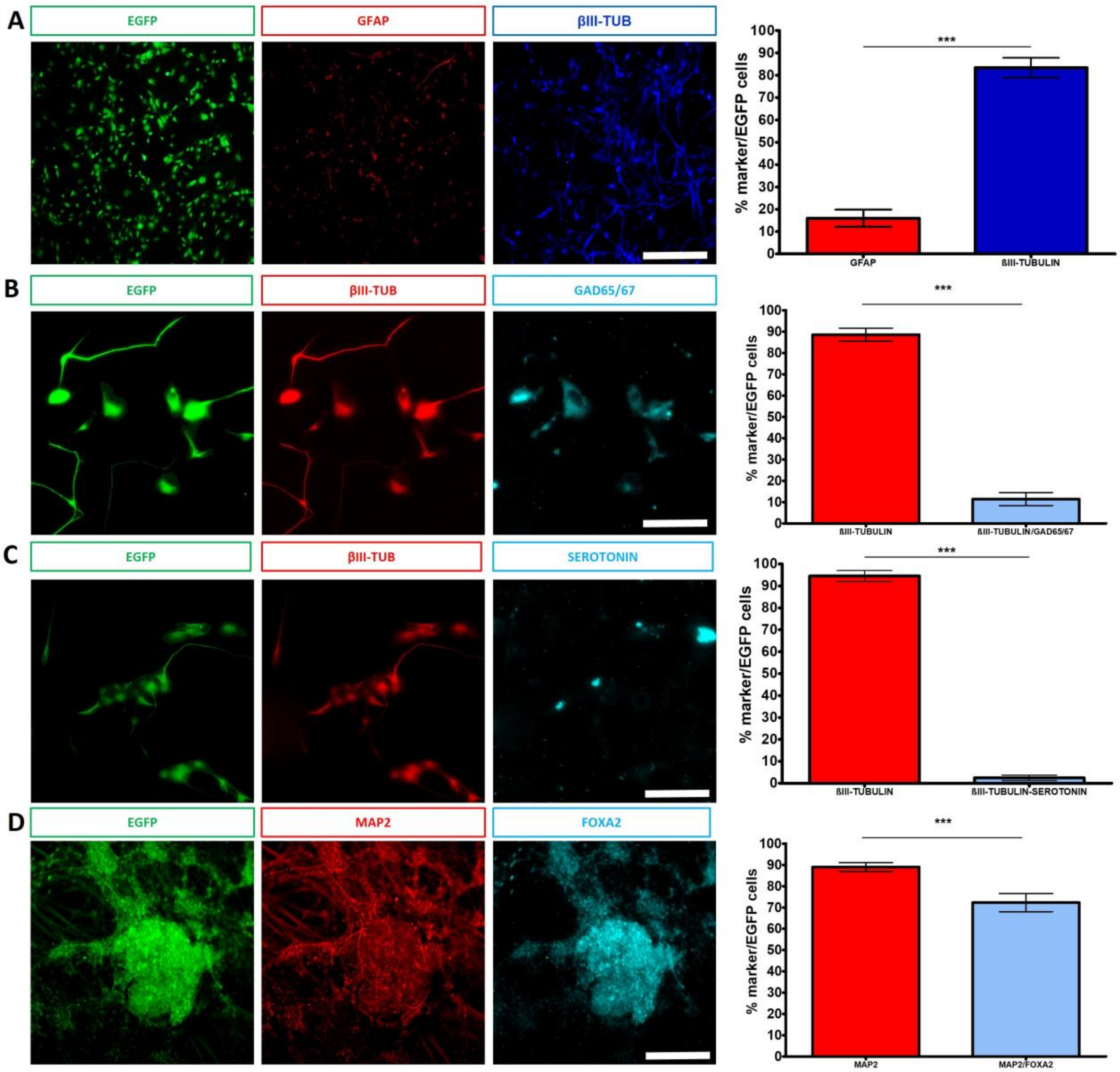


**Supplementary Figure 3.- Cell phenotypes obtained through the differentiation protocol. A)** Immunocytochemistry at day 21 for expression of EGFP, GFAP, and βIII-TUBULIN (βIII-TUB) Scale bar, 300 μm. **B and C)** Expression of βIII-TUB, GAD65/67, and Serotonin at day 28. Scale bar, 100 μm. **D)** Immunodetection of FOXA2 and MAP2 at day 35. Scale bar, 200 μm. Mean ± SEM; ****P* < 0.001, n=3 independent experiments.

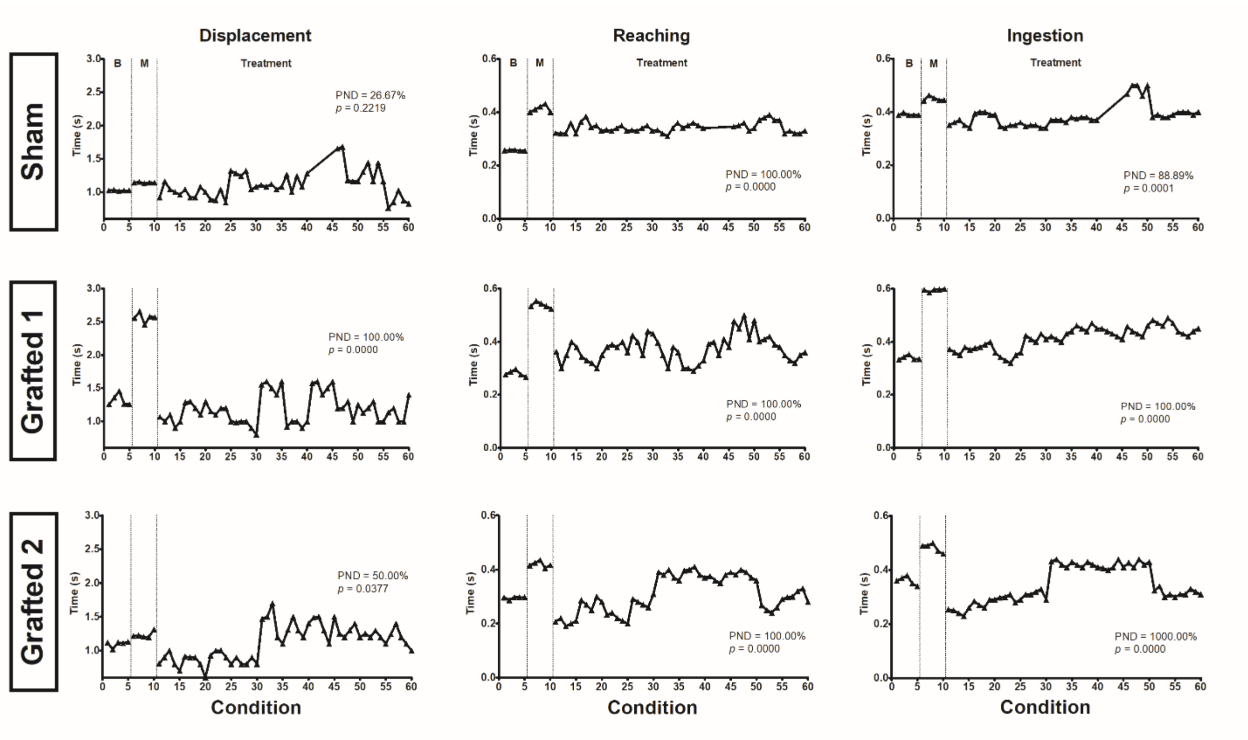


**Supplementary Figure 4.- Single-case design for each task and every NHP.** Every graph has the values for basal (B), MPTP (M), and the post-sham or -grafting surgery times up, to 10 months (Treatment) in the 3 motor tasks. The values for the 5 consecutive days of the tests are represented for B, M, and each individual month. The 3 top graphs correspond to Sham, the middle panels to Grafted 1, and the bottom are Grafted 2. The subtraction between MPTP and the average of all 10-month times was employed to compare the means (Fig 2D, bottom part). PND, Percentage of Nonoverlapping Data (Treatment vs M).

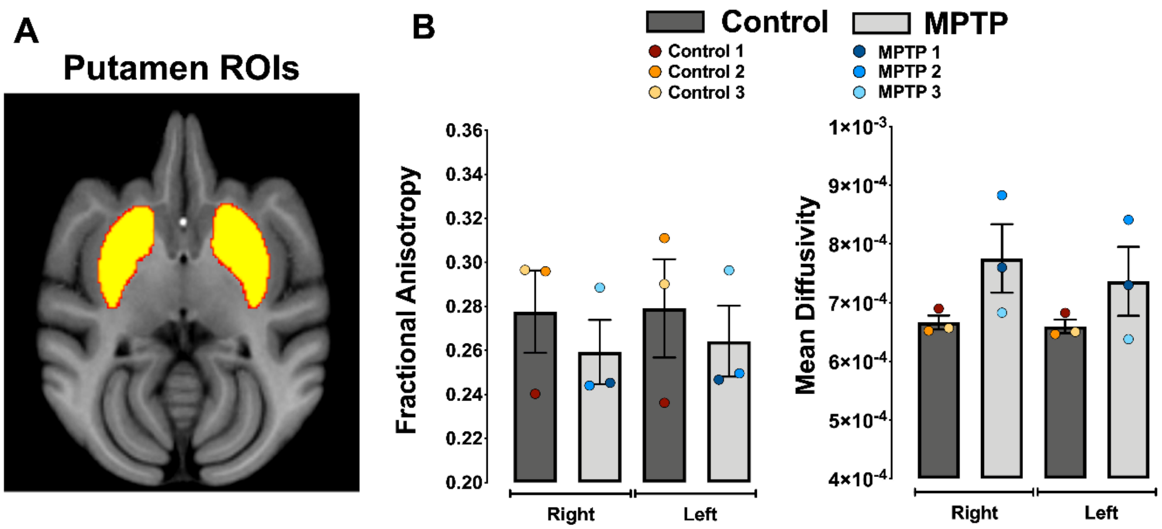


**Supplementary Figure 5.- MPTP intoxication of three NHPs diminishes Fractional Anisotropy (FA) and increases Mean Diffusivity (MD) in the putamen compared to healthy subjects. A**) MRI template and region of interest (ROI, in yellow) measured in each putamen onto anatomical brain image. FA and MD were determined bilaterally in the putamen. Three healthy NHPs were used for Control (non-parkinsonian) and three for the MPTP group. **B)** Graphs show FA and MD's comparison between the Control and MPTP-treated groups in the right and left putamina. Mean ± standard error. Two-tailed Mann-Whitney test. No significant differences were found.

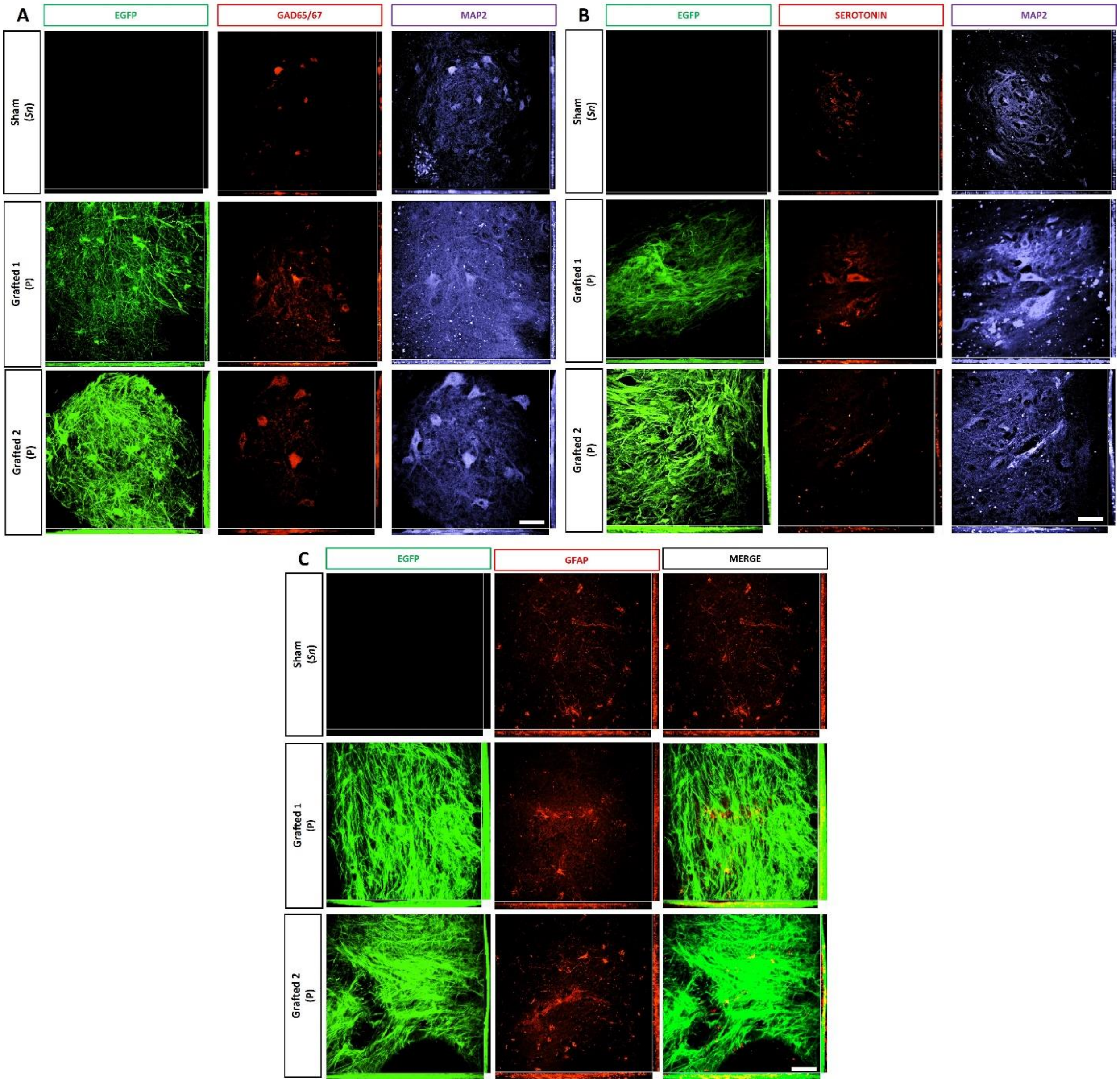


**Supplementary Figure 6.- Cell phenotypes found in the grafts.-** A) Surviving cells in the substantia nigra (Sn) and co-expression of GAD65/67 (red), MAP2 (cyan), and EGFP (green) by grafted neurons in the putamen (P) after ten months. B) Co-expression for Serotonin (red), MAP2 (cyan), and EGFP (green). C) GFAP (red) and EGFP expression. Scale bar, 50 μm.

**Supplementary Table 1.- Surgical coordinates**

| | | Sham | | | | | | Grafted 1 | | | | | | Grafted 2 | | | | | |
|---|---|---|---|---|---|---|---|---|---|---|---|---|---|---|---|---|---|---|---|
| **Graft** | | Left (cm) | | | Right (cm) | | | Left (cm) | | | Right (cm) | | | Left (cm) | | | Right (cm) | | |
| **A** | **L** | 0.9 | | | -0.95 | | | 0.9 | | | -1 | | | 1.2 | | | -1 | | |
| | **AP** | -1.85 | | | -1.85 | | | -2.2 | | | -2.2 | | | -1.3 | | | -1.3 | | |
| | **V** | -1.64 | -1.44 | -1.25 | -1.84 | -1.64 | -1.45 | -2.2 | -1.9 | -1.6 | -2.2 | -1.9 | -1.6 | -2.3 | -2 | -1.8 | -2.3 | -2 | -1.8 |
| **M** | **L** | 1.05 | | | -1.1 | | | 1 | | | -1.1 | | | 1.3 | | | -1.1 | | |
| | **AP** | -1.65 | | | -1.65 | | | -2 | | | -2 | | | -1 | | | -1 | | |
| | **V** | -1.64 | -1.44 | -1.25 | -1.84 | -1.64 | -1.45 | -2.2 | -2 | -1.8 | -2.2 | -2 | -1.8 | -2.3 | -2 | -1.8 | -2.3 | -2 | -1.8 |
| **P** | **L** | 1.2 | | | -1.25 | | | 1.1 | | | -1.2 | | | 1.4 | | | -1.3 | | |
| | **AP** | -1.36 | | | -1.36 | | | -1.8 | | | -1.8 | | | -0.8 | | | -0.8 | | |
| | **V** | -1.64 | -1.54 | -1.44 | -1.84 | -1.74 | -1.45 | -2.2 | -2 | -1.8 | -2.2 | -2 | -1.8 | -2.2 | -1.9 | -1.7 | -2.2 | -1.9 | -1.7 |
| | | | | | | | | | | | | | | | | | | | |

Coordinates are normalized to stereotaxic zero coordinates.

A, Anterior; M, Medial; P, Posterior; L, Lateral; AP; Anteroposterior; V, Ventral.

**Supplementary Table 2.- Actual *p*-values of Fig. 1A, Fig. 1F, Fig. 1H, Fig. 2D, Fig. 3D, and Fig. 6C**

**Fig. 1A**

| ***LMX1A*** | ***p*-value** |
|---|---|
| 0 vs 7 | 0.0150 |
| 0 vs 14 | 0.0001 |
| 0 vs 21 | 0.0001 |
| 0 vs 31 | 0.0001 |
| 0 vs 38 | 0.0001 |
| 7 vs 14 | 0.0150 |
| 7 vs 21 | 0.0001 |
| 7 vs 31 | 0.0001 |
| 7 vs 38 | 0.0001 |
| 14 vs 21 | 0.0080 |
| 14 vs 31 | 0.0001 |
| 14 vs 38 | 0.0001 |
| 21 vs 31 | 0.0830 |
| 21 vs 38 | 0.0420 |
| 31 vs 38 | 0.9980 |

| ***FOXA2*** | ***p*-value** |
|---|---|
| 0 vs 7 | 0.0001 |
| 0 vs 14 | 0.0001 |
| 0 vs 21 | 0.0001 |
| 0 vs 31 | 0.0001 |
| 0 vs 38 | 0.0001 |
| 7 vs 14 | 0.0330 |
| 7 vs 21 | 0.0001 |
| 7 vs 31 | 0.0001 |
| 7 vs 38 | 0.0001 |
| 14 vs 21 | 0.0180 |
| 14 vs 31 | 0.0001 |
| 14 vs 38 | 0.0001 |
| 21 vs 31 | 0.1460 |
| 21 vs 38 | 0.0180 |
| 31 vs 38 | 0.8000 |

| ***TH*** | ***p-value*** |
|---|---|
| 0 vs 7 | 1.0000 |
| 0 vs 14 | 1.0000 |
| 0 vs 21 | 0.0001 |
| 0 vs 31 | 0.0001 |
| 0 vs 38 | 0.0001 |
| 7 vs 14 | 1.0000 |
| 7 vs 21 | 0.0001 |
| 7 vs 31 | 0.0001 |
| 7 vs 38 | 0.0001 |
| 14 vs 21 | 0.0001 |
| 14 vs 31 | 0.0001 |
| 14 vs 38 | 0.0001 |
| 21 vs 31 | 0.0001 |
| 21 vs 38 | 0.0001 |
| 31 vs 38 | 0.3620 |

Comparison of the expression on different days of the differentiation protocol.

**Fig. 1F**

| **%FOXA2+ / βIII-TUB+** | ***p*-value** |
|---|---|
| 24 vs 28 | 0.0485 |
| 24 vs 35 | 0.0466 |
| 24 vs 42 | 0.0016 |
| 28 vs 35 | 0.1880 |
| 28 vs 42 | 0.2500 |
| 35 vs 42 | 0.1620 |

| **%TH+ / βIII-TUB+** | ***p-value*** |
|---|---|
| 24 vs 28 | 0.0049 |
| 24 vs 35 | 0.0042 |
| 24 vs 42 | 0.0021 |
| 28 vs 35 | 0.0880 |
| 28 vs 42 | 0.2922 |
| 35 vs 42 | 0.4600 |

Comparison of cells+ for different markers on different days of the differentiation protocol.

**Fig. 1H**

| DA release | *p*-value |
|---|---|
| B vs HK | 0.0045 |

Comparison in DA release *in vitro* in DAN at day 70 of differentiation in basal (B) solution vs isosmotic high-potassium (HK) medium.

**Fig. 2D**

| SHAM | *p*-value | | |
|---|---|---|---|
| **Conditions compared** | **Displacement** | **Reaching** | **Ingestion** |
| B vs. MPTP | 0.6268 | 0.0001 | 0.2485 |
| B vs. 1 | 0.7942 | 0.3471 | 0.8299 |
| B vs. 2 | 0.9999 | 0.0017 | 0.9999 |
| B vs. 3 | 0.4067 | 0.1114 | 0.7061 |
| B vs. 4 | 0.0021 | 0.1309 | 0.6801 |
| B vs. 5 | 0.9674 | 0.1290 | 0.9990 |
| B vs. 6 | 0.0060 | 0.0579 | 0.9999 |
| B vs. 8 | 0.0001 | 0.0344 | 0.0058 |
| B vs. 9 | 0.0002 | 0.0008 | 0.9999 |
| B vs. 10 | 0.0015 | 0.3881 | 0.9999 |
| MPTP vs. 1 | 0.0367 | 0.2688 | 0.0091 |
| MPTP vs. 2 | 0.9067 | 0.9804 | 0.6796 |
| MPTP vs. 3 | 0.0061 | 0.5147 | 0.0047 |
| MPTP vs. 4 | 0.6428 | 0.4933 | 0.0041 |
| MPTP vs. 5 | 0.9999 | 0.5143 | 0.0955 |
| MPTP vs. 6 | 0.8234 | 0.7005 | 0.1582 |
| MPTP vs. 8 | 0.0001 | 0.7848 | 0.9940 |
| MPTP vs. 9 | 0.4162 | 0.9967 | 0.2746 |
| MPTP vs. 10 | 0.0001 | 0.2462 | 0.7767 |
| 1 vs. 2 | 0.7342 | 0.9387 | 0.8111 |
| 1 vs. 3 | 0.9999 | 0.9999 | 0.9999 |
| 1 vs. 4 | 0.0001 | 0.9999 | 0.9999 |
| 1 vs. 5 | 0.2296 | 0.9999 | 0.9995 |
| 1 vs. 6 | 0.0001 | 0.9999 | 0.9974 |
| 1 vs. 8 | 0.0001 | 0.9994 | 0.0001 |
| 1 vs. 9 | 0.0001 | 0.8587 | 0.9812 |
| 1 vs. 10 | 0.5110 | 0.9999 | 0.6870 |
| 2 vs. 3 | 0.3819 | 0.9946 | 0.7018 |
| 2 vs. 4 | 0.0222 | 0.9929 | 0.6800 |
| 2 vs. 5 | 0.9979 | 0.9942 | 0.9958 |
| 2 vs. 6 | 0.0515 | 0.9996 | 0.9992 |
| 2 vs. 8 | 0.0001 | 0.9999 | 0.0917 |
| 2 vs. 9 | 0.0052 | 0.9999 | 0.9999 |
| 2 vs. 10 | 0.0025 | 0.9249 | 0.9999 |
| 3 vs. 4 | 0.0001 | 0.9999 | 0.9999 |
| 3 vs. 5 | 0.0689 | 0.9999 | 0.9971 |
| 3 vs. 6 | 0.0001 | 0.9999 | 0.9893 |
| 3 vs. 8 | 0.0001 | 0.9999 | 0.0001 |

| | | | |
|---|---|---|---|
| 3 vs. 9 | 0.0001 | 0.9759 | 0.9500 |
| 3 vs. 10 | 0.7922 | 0.9999 | 0.5600 |
| 4 vs. 5 | 0.3579 | 0.9999 | 0.9962 |
| 4 vs. 6 | 0.9999 | 0.9999 | 0.9868 |
| 4 vs. 8 | 0.0001 | 0.9999 | 0.0001 |
| 4 vs. 9 | 0.9999 | 0.9708 | 0.9422 |
| 4 vs. 10 | 0.0001 | 0.9999 | 0.5361 |
| 5 vs. 6 | 0.5431 | 0.9999 | 0.9999 |
| 5 vs. 8 | 0.0001 | 0.9999 | 0.0023 |
| 5 vs. 9 | 0.1875 | 0.9748 | 0.9999 |
| 5 vs. 10 | 0.0002 | 0.9999 | 0.9836 |
| 6 vs. 8 | 0.0001 | 0.9999 | 0.0051 |
| 6 vs. 9 | 0.9999 | 0.9960 | 0.9999 |
| 6 vs. 10 | 0.0001 | 0.9998 | 0.9956 |
| 8 vs. 9 | 0.0001 | 0.9989 | 0.0128 |
| 8 vs. 10 | 0.0001 | 0.9990 | 0.1317 |
| 9 vs. 10 | 0.0001 | 0.8362 | 0.9997 |

| **GRAFTED 1** | ***p*-value** | | |
|---|---|---|---|
| **Conditions compared** | **Displacement** | **Reaching** | **Ingestion** |
| B vs. MPTP | 0.0001 | 0.0001 | 0.0001 |
| B vs. 1 | 0.4514 | 0.4684 | 0.9564 |
| B vs. 2 | 0.9999 | 0.8345 | 0.9249 |
| B vs. 3 | 0.9709 | 0.1359 | 0.9999 |
| B vs. 4 | 0.0486 | 0.0038 | 0.0001 |
| B vs. 5 | 0.0255 | 0.0701 | 0.0654 |
| B vs. 6 | 0.0017 | 0.999 | 0.0001 |
| B vs. 7 | 0.006 | 0.073 | 0.0011 |
| B vs. 8 | 0.9998 | 0.0001 | 0.0002 |
| B vs. 9 | 0.9097 | 0.0349 | 0.0001 |
| B vs. 10 | 0.9419 | 0.7219 | 0.0058 |
| MPTP vs. 1 | 0.0001 | 0.0986 | 0.0001 |
| MPTP vs. 2 | 0.0001 | 0.0378 | 0.0001 |
| MPTP vs. 3 | 0.0001 | 0.1882 | 0.0001 |
| MPTP vs. 4 | 0.0001 | 0.7056 | 0.3424 |
| MPTP vs. 5 | 0.0001 | 0.3777 | 0.0008 |
| MPTP vs. 6 | 0.0001 | 0.004 | 0.0343 |
| MPTP vs. 7 | 0.0001 | 0.3071 | 0.0143 |
| MPTP vs. 8 | 0.0001 | 0.9976 | 0.0262 |
| MPTP vs. 9 | 0.0001 | 0.4234 | 0.1569 |
| MPTP vs. 10 | 0.0001 | 0.0448 | 0.004 |
| 1 vs. 2 | 0.4557 | 0.9999 | 0.9999 |
| 1 vs. 3 | 0.9994 | 0.9999 | 0.9979 |
| 1 vs. 4 | 0.9996 | 0.9595 | 0.0033 |
| 1 vs. 5 | 0.0001 | 0.9998 | 0.924 |
| 1 vs. 6 | 0.9159 | 0.9865 | 0.1437 |
| 1 vs. 7 | 0.0001 | 0.9999 | 0.3344 |
| 1 vs. 8 | 0.9788 | 0.2208 | 0.1856 |
| 1 vs. 9 | 0.9999 | 0.9991 | 0.0185 |
| 1 vs. 10 | 0.9999 | 0.9999 | 0.5979 |
| 2 vs. 3 | 0.9409 | 0.9993 | 0.9941 |
| 2 vs. 4 | 0.0744 | 0.7852 | 0.0082 |
| 2 vs. 5 | 0.2429 | 0.9895 | 0.9672 |
| 2 vs. 6 | 0.0055 | 0.9998 | 0.2356 |
| 2 vs. 7 | 0.1176 | 0.9938 | 0.4699 |
| 2 vs. 8 | 0.9974 | 0.075 | 0.2919 |
| 2 vs. 9 | 0.8669 | 0.9738 | 0.0394 |
| 2 vs. 10 | 0.9029 | 0.9999 | 0.7333 |
| 3 vs. 4 | 0.8686 | 0.9966 | 0.0001 |

| | | | |
|---|---|---|---|
| 3 vs. 5 | 0.0024 | 0.9999 | 0.2987 |
| 3 vs. 6 | 0.3609 | 0.868 | 0.0045 |
| 3 vs. 7 | 0.0005 | 0.9999 | 0.0201 |
| 3 vs. 8 | 0.9999 | 0.4172 | 0.0068 |
| 3 vs. 9 | 0.9999 | 0.9999 | 0.0002 |
| 3 vs. 10 | 0.9999 | 0.9998 | 0.0659 |
| 4 vs. 5 | 0.0001 | 0.9999 | 0.3987 |
| 4 vs. 6 | 0.9999 | 0.2376 | 0.9924 |
| 4 vs. 7 | 0.0001 | 0.9998 | 0.9431 |
| 4 vs. 8 | 0.6105 | 0.9781 | 0.9844 |
| 4 vs. 9 | 0.9474 | 0.9999 | 0.9999 |
| 4 vs. 10 | 0.9308 | 0.8333 | 0.7543 |
| 5 vs. 6 | 0.0001 | 0.7042 | 0.9809 |
| 5 vs. 7 | 0.9999 | 0.9999 | 0.9989 |
| 5 vs. 8 | 0.0163 | 0.7457 | 0.9903 |
| 5 vs. 9 | 0.0011 | 0.9999 | 0.7227 |
| 5 vs. 10 | 0.0017 | 0.9949 | 0.9999 |
| 6 vs. 7 | 0.0001 | 0.7392 | 0.9999 |
| 6 vs. 8 | 0.1515 | 0.0041 | 0.9999 |
| 6 vs. 9 | 0.5234 | 0.5866 | 0.9999 |
| 6 vs. 10 | 0.4836 | 0.9991 | 0.9998 |
| 7 vs. 8 | 0.0048 | 0.6425 | 0.9999 |
| 7 vs. 9 | 0.0002 | 0.9999 | 0.997 |
| 7 vs. 10 | 0.0004 | 0.9973 | 0.9999 |
| 8 vs. 9 | 0.9999 | 0.7992 | 0.9998 |
| 8 vs. 10 | 0.9999 | 0.0887 | 0.9999 |
| 9 vs. 10 | 0.9999 | 0.9854 | 0.9544 |

| GRAFTED 2 | ***p*-value** | | |
|---|---|---|---|
| **Conditions compared** | **Displacement** | **Reaching** | **Ingestion** |
| B vs. MPTP | 0.1919 | 0.1592 | 0.0003 |
| B vs. 1 | 0.0001 | 0.5592 | 0.0087 |
| B vs. 2 | 0.0001 | 0.9999 | 0.1594 |
| B vs. 3 | 0.0001 | 0.925 | 0.5386 |
| B vs. 4 | 0.0001 | 0.9999 | 0.8186 |
| B vs. 5 | 0.0001 | 0.4959 | 0.291 |
| B vs. 6 | 0.0001 | 0.3883 | 0.6234 |
| B vs. 7 | 0.0001 | 0.732 | 0.8762 |
| B vs. 8 | 0.0072 | 0.6679 | 0.3985 |
| B vs. 9 | 0.0105 | 0.9999 | 0.9794 |
| B vs. 10 | 0.0204 | 0.9999 | 0.8379 |
| MPTP vs. 1 | 0.0001 | 0.0008 | 0.0001 |
| MPTP vs. 2 | 0.0001 | 0.1733 | 0.0001 |
| MPTP vs. 3 | 0.0001 | 0.0075 | 0.0001 |
| MPTP vs. 4 | 0.0001 | 0.258 | 0.0001 |
| MPTP vs. 5 | 0.0001 | 0.9999 | 0.8279 |
| MPTP vs. 6 | 0.4834 | 0.9999 | 0.5679 |
| MPTP vs. 7 | 0.0001 | 0.9995 | 0.3186 |
| MPTP vs. 8 | 0.9981 | 0.9999 | 0.756 |
| MPTP vs. 9 | 0.9987 | 0.0834 | 0.0001 |
| MPTP vs. 10 | 0.9999 | 0.3599 | 0.0001 |
| 1 vs. 2 | 0.1731 | 0.841 | 0.9978 |
| 1 vs. 3 | 0.0911 | 0.9999 | 0.9704 |
| 1 vs. 4 | 0.3968 | 0.8146 | 0.8535 |
| 1 vs. 5 | 0.0001 | 0.0062 | 0.0001 |
| 1 vs. 6 | 0.0001 | 0.0036 | 0.0001 |
| 1 vs. 7 | 0.0001 | 0.017 | 0.0002 |
| 1 vs. 8 | 0.0001 | 0.0137 | 0.0001 |
| 1 vs. 9 | 0.0001 | 0.981 | 0.5822 |
| 1 vs. 10 | 0.0001 | 0.7562 | 0.8426 |
| 2 vs. 3 | 0.9999 | 0.9904 | 0.9999 |
| 2 vs. 4 | 0.9999 | 0.9999 | 0.9997 |
| 2 vs. 5 | 0.0001 | 0.4841 | 0.0001 |
| 2 vs. 6 | 0.0001 | 0.3878 | 0.001 |
| 2 vs. 7 | 0.0001 | 0.7015 | 0.0046 |
| 2 vs. 8 | 0.0001 | 0.6399 | 0.0003 |
| 2 vs. 9 | 0.0001 | 0.9999 | 0.9838 |
| 2 vs. 10 | 0.0001 | 0.9999 | 0.9996 |
| 3 vs. 4 | 0.9999 | 0.9851 | 0.9999 |

| | | | |
|---|---|---|---|
| 3 vs. 5 | 0.0001 | 0.0424 | 0.002 |
| 3 vs. 6 | 0.0001 | 0.0273 | 0.0105 |
| 3 vs. 7 | 0.0001 | 0.0965 | 0.0364 |
| 3 vs. 8 | 0.0001 | 0.0792 | 0.0037 |
| 3 vs. 9 | 0.0001 | 0.9999 | 0.9997 |
| 3 vs. 10 | 0.0001 | 0.9716 | 0.9999 |
| 4 vs. 5 | 0.0001 | 0.6044 | 0.0082 |
| 4 vs. 6 | 0.0001 | 0.5075 | 0.0359 |
| 4 vs. 7 | 0.0001 | 0.8012 | 0.1046 |
| 4 vs. 8 | 0.0001 | 0.7474 | 0.0143 |
| 4 vs. 9 | 0.0001 | 0.9999 | 0.9999 |
| 4 vs. 10 | 0.0001 | 0.9999 | 0.9999 |
| 5 vs. 6 | 0.0037 | 0.9999 | 0.9999 |
| 5 vs. 7 | 0.9999 | 0.9999 | 0.9998 |
| 5 vs. 8 | 0.0001 | 0.9999 | 0.9999 |
| 5 vs. 9 | 0.0001 | 0.2835 | 0.038 |
| 5 vs. 10 | 0.0001 | 0.7194 | 0.0094 |
| 6 vs. 7 | 0.0005 | 0.9999 | 0.9999 |
| 6 vs. 8 | 0.977 | 0.9999 | 0.9999 |
| 6 vs. 9 | 0.9771 | 0.2138 | 0.1274 |
| 6 vs. 10 | 0.9344 | 0.6294 | 0.0401 |
| 7 vs. 8 | 0.0001 | 0.9999 | 0.9999 |
| 7 vs. 9 | 0.0001 | 0.4714 | 0.2913 |
| 7 vs. 10 | 0.0001 | 0.8818 | 0.1144 |
| 8 vs. 9 | 0.9999 | 0.4142 | 0.0601 |
| 8 vs. 10 | 0.9999 | 0.8396 | 0.0163 |
| 9 vs. 10 | 0.9999 | 0.9999 | 0.9999 |

Comparison of the different conditions and the evaluated behaviors. B, basal; MPTP, lesioned; 1-10, month evaluated.

**Fig. 3D**

| Pearson's correlation | Reaching | Ingestion | FA-R | FA-L | MD-R | MD-L |
|---|---|---|---|---|---|---|
| **Displacement** | ***r* = 0.8980** <br> ***p* = 0.0150** | ***r* = 0.8603** <br> ***p* = 0.0278** | *r* = -0.5380 <br> *p* = 0.2707 | *r* = -0.5597 <br> *p* = 0.2480 | *r* = 0.1563 <br> *p* = 0.7673 | *r* = -0.0951 <br> *p* = 0.8577 |
| **Reaching** | | ***r* = 0.9909** <br> ***p* = 1.2e-4** | *r* = -0.6726 <br> *p* = 0.1432 | *r* = -0.3766 <br> *p* = 0.4617 | *r* = 0.2952 <br> *p* = 0.5699 | *r* = 0.0475 <br> *p* = 0.9287 |
| **Ingestion** | | | *r* = -0.7151 <br> *p* = 0.1101 | *r* = -0.4147 <br> *p* = 0.4135 | *r* = 0.3878 <br> *p* = 0.4474 | *r* = 0.1595 <br> *p* = 0.7627 |
| **FA-R** | | | | *r* = 0.5860 <br> *p* = 0.2215 | *r* = -0.5602 <br> *p* = 0.2475 | *r* = -0.5183 <br> *p* = 0.2921 |
| **FA-L** | | | | | *r* = -0.6582 <br> *p* = 0.1551 | *r* = -0.5918 <br> *p* = 0.2158 |
| **MD-R** | | | | | | ***r* = 0.9415** <br> ***p* = 0.0050** |

FA-R, Fractional anisotropy right; FA-L, Fractional anisotropy left; MD-R, Mean diffusivity right; MD-L, Mean diffusivity left.

**Fig. 6C**

| **TH+ in *Sn*** | ***p*-value** |
|---|---|
| S vs G1 | 0.0891 |
| S vs G2 | 0.0767 |

| **TH+ / EGFP+ in P** | ***p*-value** |
|---|---|
| G1 vs G2 | 0.0958 |

TH+ in the *substantia nigra* (*Sn*) and TH+/EGFP+ in the putamen (P) after grafting.

**Supplementary video legends.**

**Supplementary video S1.-** Motor behavioral assessment representative video before MPTP lesion (Sham Basal), after one month of the MPTP neurotoxic lesion (Sham MPTP administration), 4 months after surgery, and 10 months after surgery in sham NHP.

**Supplementary video S2.-** Motor behavioral assessment representative video before MPTP lesion (Grafted 1 Basal), after one month of the MPTP neurotoxic lesion (Grafted 1 MPTP administration), 4 months after surgery, and 10 months after surgery in Grafted 1 NHP.

**Supplementary video S3.-** Motor behavioral assessment representative video before MPTP lesion (Grafted 2 Basal), after one month of the MPTP neurotoxic lesion (Grafted 2 MPTP administration), 4 months after surgery, and 10 months after surgery in Grafted 2 NHP.
